# EZH2–CCF–cGAS Axis Promotes Breast Cancer Metastasis

**DOI:** 10.3390/ijms23031788

**Published:** 2022-02-04

**Authors:** Dandan Duan, Mengjie Shang, Yanxu Han, Jiayuan Liu, Jiwei Liu, Sun Hyok Kong, Jingyao Hou, Baiqu Huang, Jun Lu, Yu Zhang

**Affiliations:** 1 The Key Laboratory of Molecular Epigenetics of Ministry of Education (MOE), Northeast Normal University, Changchun 130024, China; duandd305@nenu.edu.cn (D.D.); shangmj340@nenu.edu.cn (M.S.); hanyanxuxu@126.com (Y.H.); kongsg354@nenu.edu.cn (S.H.K.); luj809@nenu.edu.cn (J.L.); 2 The Institute of Genetics and Cytology, Northeast Normal University, Changchun 130024, China; liu18804225466@163.com (J.L.); houjy866@nenu.edu.cn (J.H.); huangbq@nenu.edu.cn (B.H.); 3 School of Life Science and Technology, Inner Mongolia University of Science & Technology, Baotou 014010, China; 2020904@imust.edu.cn

**Keywords:** breast cancer metastasis, cytoplasmic chromatin fragments, EZH2, cGAS, USP7

## Abstract

Cytoplasmic chromatin fragments (CCF) are recognized by the cytoplasmic DNA sensor cyclic GMP-AMP synthase (cGAS), which activates the cGAS–STING (cyclic GMP-AMP synthase-stimulator of interferon genes) pathway and promotes the production of inflammatory factors and breast cancer metastasis. However, the mechanisms by which CCF are formed in tumor cells and CCF activation cGAS promotes breast cancer metastasis remain unclear. Here, we report that the enhancer of zeste homolog 2 (EZH2) can promote the formation of CCF and activate the cGAS–STING pathway to promote breast cancer metastasis. Further research found that the EZH2-mediated CCF formation depended on high mobility group A1 (HMGA1), while the stability of EZH2 required ubiquitin-specific peptidase 7 (USP7), indicating that the EZH2–HMGA1–USP7 complex regulated CCF formation. Moreover, EZH2 can activate cGAS through CCF, requiring USP7 to deubiquitinate cGAS and stabilize cGAS. In vivo experimental results showed that EZH2 could promote breast cancer metastasis through CCF. Our findings highlight a new target for breast cancer metastasis. Targeting the EZH2–CCF–cGAS axis may be a potential therapeutic strategy for inhibiting breast cancer metastasis.

## 1. Introduction

Breast cancer seriously threatens women’s health, and breast cancer metastasis is the main cause of death in patients with this disease [1]. Recently, tumor cells have been reported to contain cytoplasmic chromatin fragments (CCF), a DNA complex found in the micronucleus of cells [2,3,4,5]. The main components of CCF include genomic DNA, γH2AX, and the heterochromatin markers H3K9me3 and H3K27me3. However, the lack of some euchromatin markers suggests that CCF may be derived from heterochromatin regions that are transcriptionally repressed [3]. Studies have demonstrated that CCF can promote cell senescence, the senescence-associated secretory phenotype, and inflammatory factor secretion, as well as the occurrence and development of tumors [3,6,7]. However, the mechanism by which CCF is formed is far from elucidated. GMP-AMP synthase (cGAS) is a recently identified type of cytoplasmic DNA sensor found in mammalian cells [8]. Studies have revealed that tumor cells with high chromosomal instability (CIN) can activate the cGAS–STING (cyclic GMP-AMP synthase-stimulator of interferon genes) pathway through cytoplasmic DNA to produce inflammatory factors, thereby promoting tumor metastasis [7]. CCF is a unique type of cytoplasmic DNA, and it has been reported that CCF can also activate the cGAS–STING pathway to promote the production of inflammatory factors [3]. Therefore, studying the mechanism by which CCF activates the cGAS–STING pathway will help identify new targets for the treatment of tumors.

Enhancer of zeste homolog 2 (EZH2) is the histone methyltransferase of H3K27, which catalyzes the trimethylation of histone H3K27, promotes the formation of heterochromatin, and mediates the transcriptional silencing of its downstream target genes [9,10,11]. In addition, EZH2 can promote the secretion of inflammatory factors, regulate the cell cycle and cell proliferation, and promote the occurrence and development of cancer [12,13,14]. Studies have demonstrated that EZH2 is significantly up-regulated in various malignancies, including esophageal cancer, breast cancer, gastric cancer, anaplastic thyroid cancer, nasopharyngeal cancer, and endometrial cancer [10,14,15,16]. EZH2 is positively correlated with breast cancer metastasis, invasion, and angiogenesis, and it is a biomarker of invasive breast cancer [12,17,18]. The relationship between EZH2 and CCF in the cGAS–STING pathway is still unclear. We speculate that EZH2 participates in CCF formation and the activation of the cGAS–STING pathway.

It has been reported that the ubiquitination of cGAS is correlated with the activation of cGAS [19,20]. Studies have demonstrated that deubiquitinating enzymes, such as Ubiquitin-specific peptidase 14 (USP14) and Ubiquitin-specific peptidase 27X (USP27X), can affect the cGAS protein level [21,22], but the effect of USP7 on cGAS expression has not yet been reported. Ubiquitin-specific peptidase 7 (USP7) is a deubiquitinating enzyme, and it has been revealed that many tumorigenesis-promoting proteins are also substrates of USP7. USP7 overexpression correlates with various malignancies, including epithelial cancer, prostate cancer, breast cancer, lung cancer, and cervical cancer, through the regulation of cancer-promoting or tumor-suppressing proteins [23,24,25,26]. Furthermore, it has been reported that EZH2 can directly bind to USP7 in prostate cancer cells to stabilize EZH2, thereby promoting metastasis [27,28]. Therefore, we speculate that USP7 may also be involved in the formation of CCF and the activation of cGAS.

The purpose of this study is the mechanism of CCF formation and the activation of the cGAS-STING pathway, which will help people develop a new strategy for breast cancer. Our data show that EZH2 can promote CCF formation, and EZH2 activates the cGAS–STING pathway through CCF to promote breast cancer metastasis. Further experiments revealed that the EZH2-mediated CCF formation depends on HMGA1, while EZH2 stability requires USP7, indicating that the EZH2–HMGA1–USP7 complex stabilizes CCF. Moreover, EZH2 activates cGAS through CCF, requiring USP7 to deubiquitinate cGAS and stabilize cGAS. Therefore, the EZH2–CCF–cGAS axis can be targeted in breast cancer metastasis, and new targets of this pathway may be potential therapeutic strategies to inhibit breast cancer metastasis.

## 2. Results

### 2.1. EZH2 Promotes the Formation of CCF in Breast Cancer Cells

**Figure 1:**
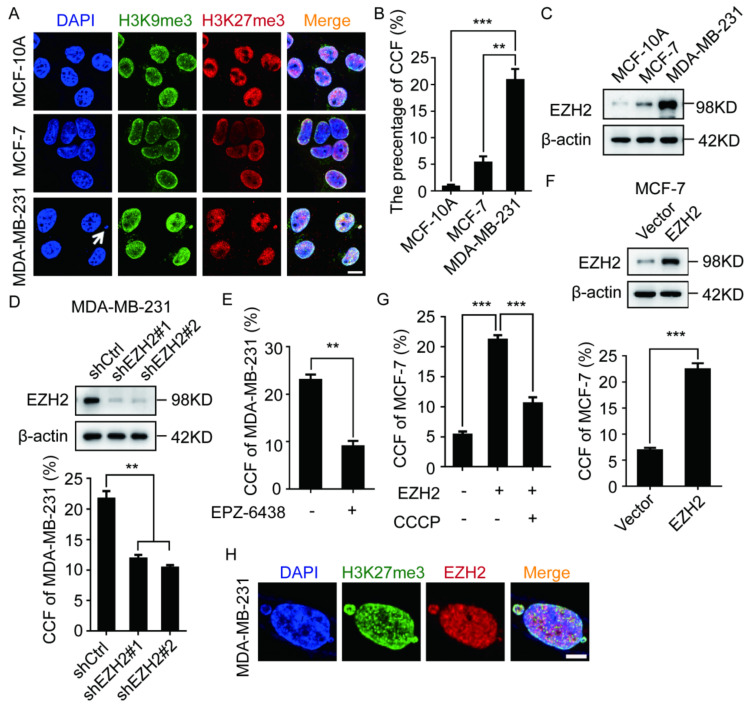
EZH2 participates in the formation of CCF in breast cancer cells. (**A**,**B**) MCF-10A, MCF-7, MM-231 cells were immunofluorescent stained with H3K9me3 and H3K27me3 to calculate the ratio of CCF. CCF was indicated by arrows. Scale bars = 10 μm. Western blotting was used to detect the EZH2 level in MCF-10A, MCF-7, MM-231 (**C**), MM-231-shCtrl/MM-231-shEZH2 (**D**), MCF-7-EZH2 cells (**F**). The ratio of CCF was calculated by immunofluorescence in MM-231-shCtrl/MM-231-shEZH2 cells (**D**) MM-231 cells treated with EPZ-6438 (1 μM) for 72 h (**E**), MCF-7-EZH2 cells (**F**), MCF-7-EZH2 cells treated with CCF inhibitor CCCP (50 μM) for 2 h (**G**). H EZH2, H3K27me3 were immunofluorescent stained in MM-231 cells to observe the co-localization of EZH2 with CCF. Scale bars = 5 μm. Each experiment was repeated at least 3 times. Error bars, mean ± SD, **, *p* < 0.01; ***, *p* < 0.001.

Studies have shown that CCF has the characteristics of heterochromatin, and its key markers are H3K9me3 and H3K27me3 [3]. We examined H3K9me3 localization in different tumor cell lines and observed that CCF existed in different tumor cell lines (Figure S1A), and the proportion of CCF was the highest in breast cancer MDA-MB-231 (MM-231) cells (Figure S1B). Therefore, we decided to choose breast cancer cells for further study. We labeled with H3K9me3 and H3K27me3 in MCF-10A, MCF-7, and MM-231 cells and observed that the proportion of CCF was the highest in highly metastatic MM-231 cells (Figure 1A,B). Studies have demonstrated that adriamycin-resistant MCF-7 (MCF-7/ADR) breast cancer cells are highly metastatic [29]. To confirm that the proportion of CCF in highly metastatic breast cancer cells was increased, we labeled H3K9me3 and H3K27me3 in MCF-7 and MCF-7/ADR cells (Figure S2A). The results showed that CCF proportion in MCF-7/ADR cells was significantly increased (Figure S2B).

As H3K27me3 is a marker of CCF [3], EZH2 is an enzyme that catalyzes the modification of H3K27me3 and promotes the formation of heterochromatin [30]. We speculated that EZH2 may be involved in CCF formation. We compared the EZH2 level in various breast cancer cells and observed that the EZH2 level in MM-231 and MCF-7/ADR cells were higher than that in MCF-10A and MCF-7 cells (Figure 1C and Figure S2C). Therefore, we labeled H3K27me3 in MM-231-shEZH2 and MCF-7/ADR-shEZH2 cells. The results showed that EZH2 knockdown could significantly inhibit CCF formation (Figure 1D and Figure S2D). To confirm these results, we also labeled H3K27me3 in MM-231 and MCF-7/ADR cells treated with the EZH2 inhibitor EPZ-6438, and we observed that EPZ-6438 could significantly inhibit CCF formation (Figure 1E and Figure S2E).

We then labeled H3K27me3 in MCF-7-EZH2 cells and observed that EZH2 overexpression could significantly promote CCF formation (Figure 1F). It has been reported that CCCP (Carbonyl cyanide m-chlorophenyl hydrazone) is a CCF scavenger [31]. However, after adding CCCP to MCF-7-EZH2 cells, the proportion of CCF was significantly reduced (Figure 1G). In localization experiments of EZH2 and H3K27me3, almost all EZH2 was colocalized with CCF in MM-231 and MCF-7/ADR cells (Figure 1H and Figure S2F,G). These results indicate that EZH2 can promote CCF formation in MCF-7 cells.

### 2.2. EZH2 Activates the cGAS–STING Pathway through CCF

**Figure 2:**
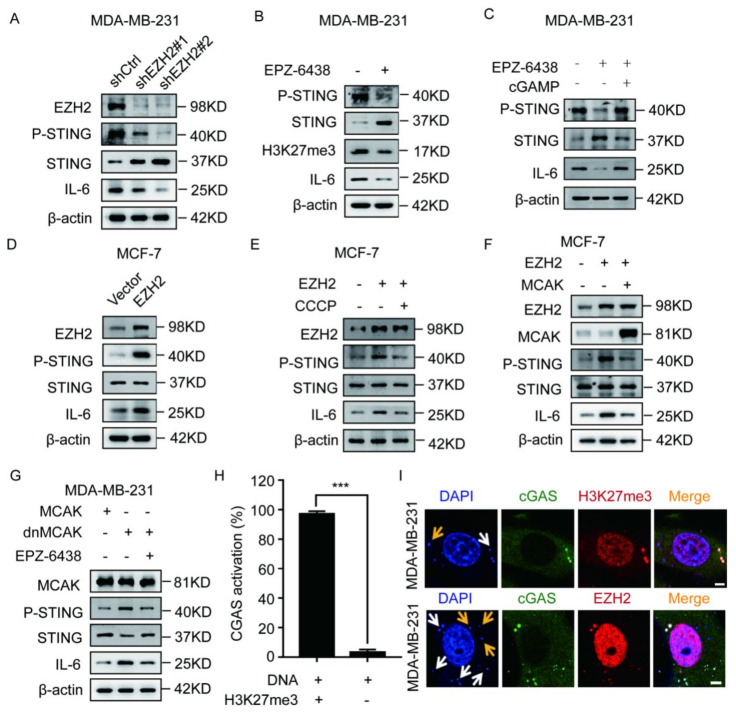
EZH2 affects the activation of the cGAS-STING pathway through CCF in breast cancer cells. Western blotting was used to detect the P-STING, STING, and IL-6 level in MM-231-shCtrl/MM-231-shEZH2 cells (**A**), MM-231 cells treated with EPZ-6438 (1 μM) for 72 h (**B**), MM-231 cells treated with cGAMP (5 μM) for 4 h (**C**), MCF-7-EZH2 cells (**D**), MCF-7-EZH2 cells treated with the CCF inhibitor CCCP (**E**), MCF-7-EZH2 cells overexpressing MCAK (**F**), MM-231-dnMCAK cells treated with EPZ-6438 (1 μM) for 72 h (**G**). (**H**–**I**) MM-231 cells were immunofluorescent stained with DAPI, H3K27me3, EZH2, and cGAS to calculate the ratio and observe cGAS activated by CCF (white arrow) or naked DNA (yellow arrow). Meanwhile, we observe whether there is EZH2 in the activated cGAS, scale bar = 5 μm. Each experiment was repeated at least three times. Error bars, mean ± SD, ***, *p* < 0.001.

As CCF activates the cGAS–STING pathway [3], we examined if EZH2 can affect the activation of the cGAS–STING pathway. Compared with controls, p-STING and IL-6 levels in MM-231-shEZH2, MCF-7/ADR-shEZH2, MM-231, and MCF-7/ADR cells treated with EPZ-6438 were significantly down-regulated (Figure 2A,B and Figure S3A,B). It has been reported that cGAS activation promotes cyclin GMP-AMP (cGAMP) production, while the exogenous cGAMP can restore the activation of cGAS downstream pathways when the cGAS activity is inhibited [7,32]. Therefore, we treated MM-231 cells in the presence of cGAMP and EPZ-6438 and observed that cGAMP could restore p-STING and IL-6 levels (Figure 2C). The levels of p-STING and IL-6 in MCF-7-EZH2 cells were also significantly up-regulated (Figure 2D). These results indicate that EZH2 can promote the activation of the cGAS–STING pathway.

Next, we examined if EZH2 can promote the cGAS–STING pathway activation through CCF. Our results showed that p-STING and IL-6 levels were significantly suppressed after treatment of MCF-7-EZH2 cells with CCCP (Figure 2E). Previous studies have demonstrated that CIN (chromosome instability) can promote cytoplasmic DNA formation. MCAK (mitotic centromere-associated kinesin) overexpression can decrease CIN, thereby reducing the cytoplasmic DNA levels, while dnMCAK (dominant-negative MCAK mutant) overexpression can increase CIN, further increasing cytoplasmic DNA levels [7]. We found that p-STING and IL-6 protein levels were down-regulated after MCAK was overexpressed in MCF-7-EZH2 cells (Figure 2F). In addition, compared with MM-231-MCAK cells, p-STING and IL-6 levels were up-regulated in MM-231-dnMCAK cells, while treatment of MM-231-dnMCAK cells with EPZ-6438 down-regulated p-STING and IL-6 levels (Figure 2G). These results show that EZH2 can promote the cGAS–STING pathway activation through CCF.

Cytoplasmic DNA is generally considered to consist of fragments of naked DNA, while CCF is considered to consist of fragments of heterochromatin that belong to a unique type of cytoplasmic DNA. Therefore, we checked which type of cytoplasmic DNA activates cGAS in MM-231 cells. We labeled nuclei with DAPI and cGAS, H3K27me3, and EZH2 proteins in MM-231 cells and observed that 90% of cGAS was activated by CCF (Figure 2H). Among these, almost all cGAS was colocalized with CCF and EZH2 in MM-231 cells (Figure 2I). These results indicate that cGAS is mainly activated by CCF in MM-231 cells. 

### 2.3. The EZH2-Mediated CCF Formation Depends on HMGA1

**Figure 3:**
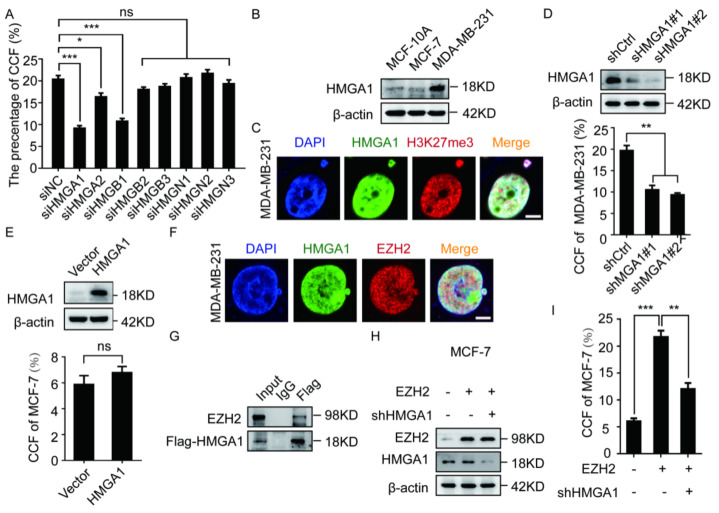
HMGA1 is a necessary condition for EZH2 to promote the formation of CCF. (**A**) Interfering with the HMG family of MM-231 cells were immunofluorescent stained with H3K27me3 to calculate the ratio of CCF. Western blotting was used to detect the HMGA1 level in MCF-10A, MCF-7, MM-231 (**B**) MM-231-shCtrl/MM-231-shHMGA1 (**D**) MCF-7-HMGA1 cells (**E**). (**C**) MM-231 cells were immunofluorescent stained with HMGA1 and H3K27me3 to observe the co-localization of HMGA1 and EZH2, Scalebars = 5 μm. The ratio of CCF was calculated by immunofluorescence in MM-231-shCtrl/MM-231-shHMGA1 (**D**), MCF-7-HMGA1 cells (**E**). (**F**) MM-231 cells were immunofluorescent stained with HMGA1 and EZH2 to observe the co-localization of HMGA1 and EZH2, Scalebars = 5 μm. (**G**) After 48 h of transient transfection of EZH2 and HMGA1 overexpression constructs in HEK-293T cells, the Flag antibody was used for co-IP. Western blotting was used to detect the EZH2 and HMGA1 level in HEK-293T (**G**), MCF-7-EZH2 cells knocked down HMGA1 (**H**). (**I**) Calculate the ratio of CCF in (**H**). Each experiment was repeated at least 3 times. Error bars, mean ± SD, *, *p* < 0.05; **, *p* < 0.01; ***, *p* < 0.001.

It has been reported that EZH2 cannot directly bind to DNA [33], while HMG family proteins can maintain chromatin structure [34]. In order to further study the mechanism by which EZH2 promotes CCF formation, we screened HMG family proteins to test whether they can affect CCF formation. We silenced the expression of the main members of the HMG family, HMGA1/2, HMGB1/2/3, and HMGN1/2/3, and labeled H3K9me3 and H3K27me3 in MM-231 cells. The results indicated that HMGA1 and HMGB1 knockdown could significantly inhibit CCF formation (Figure 3A and Figure S4A).

HMGA1 functions to maintain chromatin structure and directly binds to DNA [20,35,36]. To confirm the role of HMGA1 in CCF formation, we examined the HMGA1 level in a variety of breast cancer cells and observed that the HMGA1 level in MM-231 and MCF-7/ADR cells was higher than that in MCF-10A and MCF-7 cells (Figure 3B and Figure S4B). In addition, we labeled HMGA1 and H3K27me3 in MM-231 and MCF-7/ADR cells and observed that almost all HMGA1 was colocalized with CCF (Figure 3C and Figure S4C,D). For further verification, we labeled H3K27me3 in MM-231-shHMGA1 cells. HMGA1 knockdown could significantly inhibit CCF formation (Figure 3D). In addition, we labeled H3K27me3 in MCF-7-HMGA1 cells and observed that the proportion of CCF in cells overexpressing HMGA1 did not change significantly (Figure 3E), indicating that HMGA1 alone cannot promote the formation of CCF. As such, we conclude that HMGA1 is a necessary but not sufficient condition for CCF formation.

Next, we labeled HMGA1 and EZH2 in MM-231 cells and observed that HMGA1 was colocalized with EZH2 within CCF (Figure 3F and Figure S4E). Co-IP results revealed the interaction between HMGA1 and EZH2 (Figure 3G). In addition, we labeled H3K27me3 in HMGA1-silenced MCF-7-EZH2 cells and observed that HMGA1 knockdown decreased the CCF ratio (Figure 2H,I). These results indicate that HMGA1 is necessary for EZH2-mediated CCF formation. 

### 2.4. The EZH2–HMGA1–USP7 Complex Stabilizes CCF

**Figure 4:**
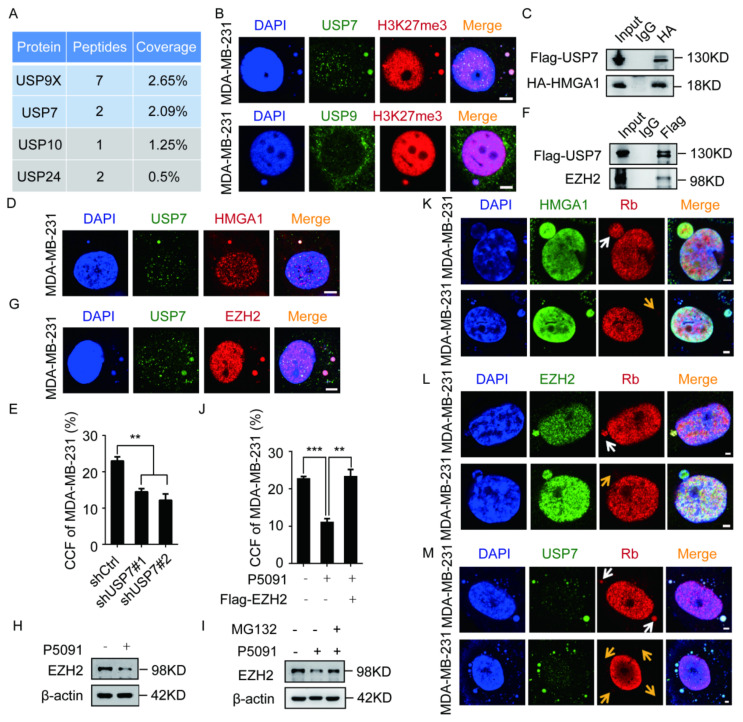
USP7 and HMGA1 stabilize CCF by stabilizing EZH2. (**A**) Representative results of Flag-HMGA1 combined with mass spectrometry in HEK-293T cells. (**B**) USP7, H3K27me3, and USP9X were immunofluorescent stained in MM-231 cells to observe the co-localization of USP7 and USP9X with CCF. (**C**,**F**) After 48 h of transient transfection of HMGA1, EZH2 and USP7 overexpression constructs in HEK-293T cells, the HA/Flag antibody was used for co-IP. Western blotting was used to detect the interaction between HMGA1 and EZH2 with USP7. (**D**,**G**) USP7, HMGA1, EZH2 were immunofluorescent stained in MM-231 cells to observe the co-localization of HMGA1 and EZH2 with USP7. The ratio of CCF was calculated by immunofluorescence in MM-231-shCtrl/MM-231-shUSP7 (**E**), restoring EZH2 levels of MM-231 cells (**J**). Western blotting was used to detect the EZH2 level in MM-231 cells treated with P5091 (10 μM) for 48 h (**H**), MM-231 cells treated with MG132 (10 μM) for 12 h (**I**). (**K**–**M**) MM-231 cells were immunofluorescent stained with HMGA1 and Rb, EZH2 and Rb, and USP7 and Rb to detect the presence of HMGA1, EZH2, and USP7 before (white arrow) or after (yellow arrow) the rupture of the micronuclear membrane. Scale bars = 2 μm. Each experiment was repeated at least 3 times. Error bars, mean ± SD. **, *p* < 0.01; ***, *p* < 0.001.

Multiple deubiquitinates were identified in the earlier HMGA1-binding mass spectrometry in our laboratory. In order to study the relationship between deubiquitinating enzymes and CCF, We selected the higher abundance USP7 and Ubiquitin-specific peptidase 9 (USP9) to observe the co-localization with CCF by immunofluorescence and found that USP7 and CCF colocalized (Figure 4B). The co-IP results confirmed that USP7 and HMGA1 were present in the same complex (Figure 4C). In addition, USP7 and HMGA1 were labeled in MM-231 cells, and USP7 was observed to be colocalized with HMGA1 (Figure 4D). Next, we labeled H3K27me3 in MM-231-shUSP7 cells and observed that USP7 knockdown could inhibit CCF formation (Figure 4E). Therefore, we concluded that HMGA1 binds to USP7, thereby stabilizing CCF.

It has been reported that EZH2 recruits USP7 to stabilize EZH2 in prostate cancer cells [27]. We used co-IP to confirm the interaction between EZH2 and USP7 (Figure 4F). Next, we labeled USP7 and EZH2 in MM-231 cells and observed that USP7 colocalized with EZH2 (Figure 4G). When MM-231 cells were treated with the USP7 inhibitor P5091, the EZH2 protein level decreased (Figure 4H); however, it was restored by the addition of MG132 (Z-Leu-Leu-Leu-al) (Figure 4I). After treatment with P5091 and MG132, we labeled H3K27me3 in MM-231 cells and observed that the increased EZH2 level restored the proportion of CCF (Figure 4J). These results show that USP7 stabilizes CCF by stabilizing EZH2. Studies have found that CCF in tumor cells is mainly derived from micronuclei. Rb is an indicator of the integrity of the micronucleus membrane [37]. Therefore, we labeled the endogenous nuclear proteins retinoblastoma (Rb) and HMGA1/EZH2/USP7 in MM-231 cells and observed HMGA1/EZH2/USP7 both before and after the rupture of the micronucleus membrane (Figure 4K–M). We concluded that the EZH2–HMGA1–USP7 complex stabilizes CCF. 

### 2.5. USP7 Stabilizes and Activates cGAS

**Figure 5:**
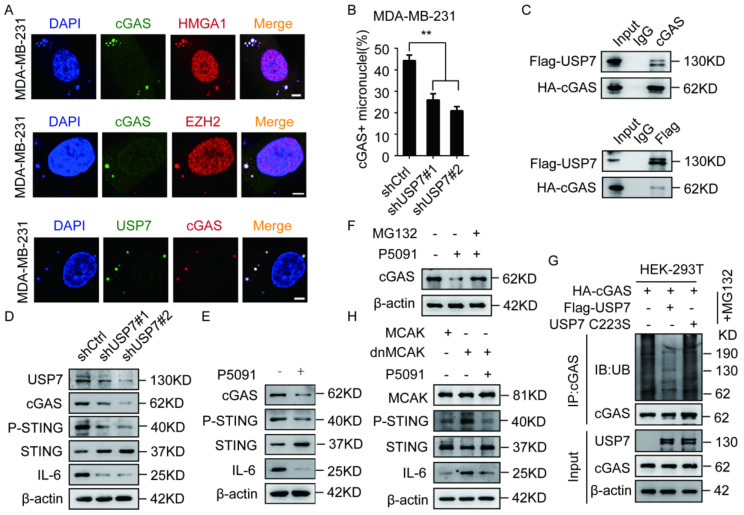
USP7 stabilizes cGAS and activates the cGAS-STING pathway. (**A**) MM-231 cells were immunofluorescent stained with HMGA1, EZH2, USP7, and cGAS to observe the co-localization of HMGA1, EZH2, and USP7 with cGAS, Scalebars = 5 μm. (**B**) MM-231-shUSP7 cells were immunofluorescent stained with cGAS and H3K27me3 to calculate the ratio of cGAS activation. (**C**) After 48 h of transient transfection of USP7 and cGAS overexpression constructs in HEK-293T cells, the Flag antibody was used for co-IP. Western blotting was used to detect the USP7, cGAS, P-STING, STING, IL-6 level in HEK-293T (**C**), MM-231-shCtrl/MM-231-shUSP7 (**D**), MM-231 cells treated with P5091 (10 μM) for 48 h (**E**), MM-231 cells treated with MG132 (10 μM) for 10 h (**F**), MM-231-dnMCAK cells treated with P5091 (10 μM) for 48 h (**H**). (**G**) HEK-293T cells were transfected with USP7 WT or USP7 C223S, then treated with MG132 (10 μM) for 10 h. Cell lysates were immunoprecipitated using an anti-HA antibody followed by immunoblotting analysis. Each experiment was repeated at least 3 times. Error bars, mean ± SD. **, *p* < 0.01.

Because CCF can activate cGAS [3], we examined if the EZH2–HMGA1–USP7 complex can be colocalized with cGAS. Our results showed that the EZH2–HMGA1–USP7 complex was colocalized with cGAS (Figure 5A). It has been reported that the ubiquitination of cGAS is correlated with the activation of cGAS [19], but the relationship between USP7 and cGAS has not yet been investigated. We labeled DAPI and cGAS in MM-231-shUSP7 cells and observed that USP7 knockdown could inhibit cGAS activation (Figure 5B). In addition, co-IP results confirmed the interaction between USP7 and cGAS (Figure 5C). We found that USP7 knockdown or P5091 treatment of MM-231 cells down-regulated p-STING and IL-6 levels (Figure 5D,E). These results indicate that USP7 can affect the activation of the cGAS–STING pathway.

Studies have shown that deubiquitinating enzymes, such as USP14 and USP27X, can affect the cGAS protein level [21,22], but the role of USP7 in the regulation of cGAS expression has not yet been reported. We observed that USP7 knockdown or P5091 treatment of MM-231 cells could inhibit the cGAS protein level (Figure 5D,E), and MG132 treatment could restore the cGAS protein level (Figure 5F). Since USP7 is a deubiquitinating enzyme, we tested the effect of USP7 on the ubiquitination level of cGAS. We found that overexpression of USP7-WT, but not catalytically inactive USP7-MT (C223S), significantly lowered the ubiquitination of cGAS in HEK293T cells (Figure 5G). Since CCF can activate cGAS, and our study found that USP7 affected cGAS activation, we want to study whether USP7 is required for CCF to activate cGAS. When MM-231 cells overexpressing dnMCAK were treated with P5091, p-STING and IL-6 levels were inhibited (Figure 5H). These results indicate that USP7 stabilizes cGAS and activates the cGAS-STING pathway.

### 2.6. EZH2 Promotes Breast Cancer Metastasis through CCF

**Figure 6:**
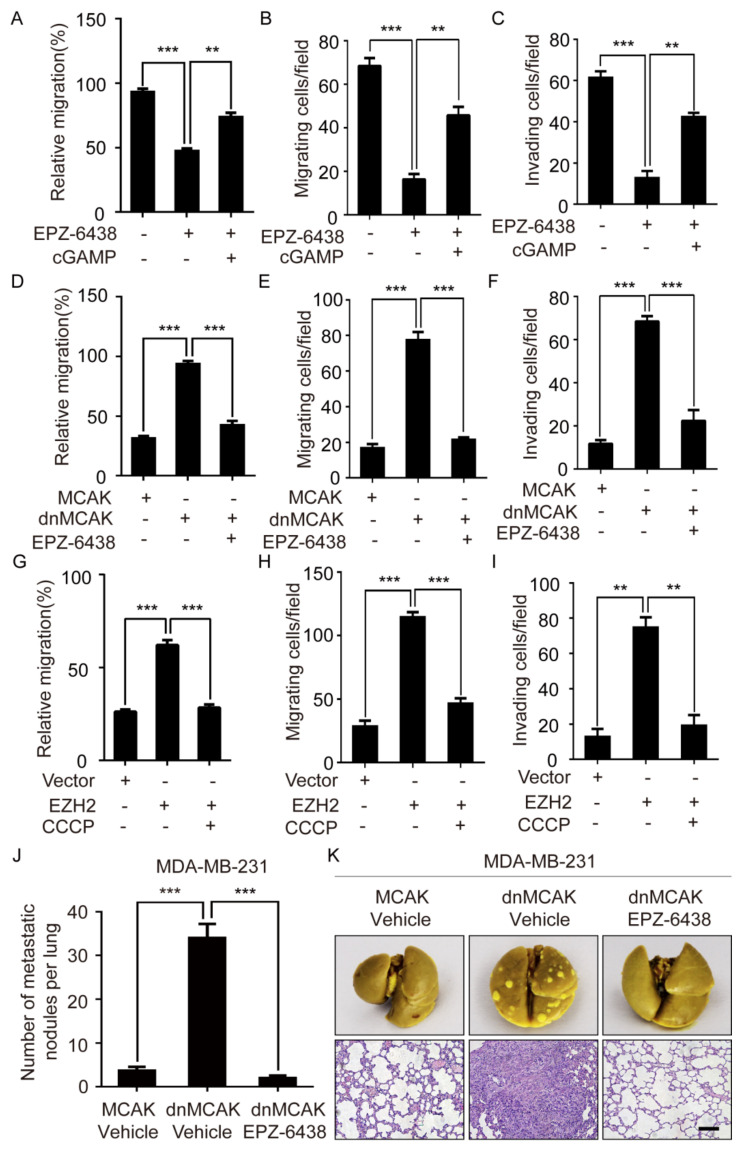
EZH2 promotes the increase of lung metastases in mice through CCF. Wound-healing assay of MM-231 cells was treated with EPZ-6438 and cGAMP (**A**), MM-231-dnMCAK cells treatment with EPZ-6438 (**D**), MCF-7-EZH2 cells treatment with CCCP (**G**). Migration assays of MM-231 cells were treated with EPZ-6438 and cGAMP (**B**), MM-231-dnMCAK cells treatment with EPZ-6438 (**E**), MCF-7-EZH2 cells treatment with CCCP (**H**). Invasion assays of MM-231 cells were treated with EPZ-6438 and cGAMP (**C**), MM-231-dnMCAK cells treatment with EPZ-6438 (**F**), MCF-7-EZH2 cells treatment with CCCP (**I**). (**J**) Nude mice were injected with a certain number of MM-231-MCAK and MM-231-dnMCAK cells in the tail vein. One week later, EPZ-6438 (34 mg/kg) was injected into the dnMCAK group, and the other two groups were injected with a vehicle for two consecutive weeks. After 4 weeks, the mice were sacrificed, and the lung tissues were dissected and fixed with Bouin’s fixative. The macroscopic metastases on the surface of the fixed lung tissues were counted, and the differences were analyzed. Error bars, mean ± SD. ***, *p* < 0.001. (**K**) The mouse lung tissue was stained with HE to observe the metastasis. Scale bars = 100 μm. Each experiment was repeated at least 3 times. Error bars, mean ± SD. **, *p* < 0.01; ***, *p* < 0.001.

To study whether EZH2 promotes breast cancer cell migration and invasion through cGAS, we treated MM-231 cells with EPZ-6438 and observed that EPZ-6438 impaired cell migration and invasion. After treating MM-231 cells with EPZ-6438 and cGAMP, the migration and invasion of cells were restored and compared with that of the EPZ-6438 group (Figure 6A–C and Figure S5A). 

Because CCF can activate cGAS, we next investigated if EZH2 can promote breast cancer cell migration and invasion through CCF. The results showed that the migration and the invasion of MM-231-dnMCAK cells were enhanced compared with that of the MM-231-MCAK cells. At the same time, the migratory and invasive abilities of MM-231-dnMCAK cells treated with EPZ6438 were inhibited (Figure 6D–F and Figure S5B). Furthermore, the migratory and invasive abilities of MCF-7-EZH2 cells were enhanced compared with that of the MCF-7 cells, while both abilities in MCF-7-EZH2 cells treated with CCCP were weakened (Figure 6G–I and Figure S5C). These results indicate that EZH2 can promote the migration and the invasion of breast cancer cells through CCF. 

To further verify that EZH2 can promote breast cancer metastasis through CCF in vivo, MM-231 cells overexpressing MCAK (decreased CCF) and dnMCAK (increased CCF) were injected into the tail veins of female nude mice. One week later, EPZ-6438 was injected intraperitoneally into the dnMCAK group mice daily for two consecutive weeks, and mice were maintained for another 4 weeks without any further treatment. Thereafter, lung tissues were harvested and examined by hematoxylin-eosin staining. Mice injected with MCAK cells formed fewer lung metastatic foci than those injected with dnMCAK cells. In addition, the metastasis of mice injected with EPZ-6438 was significantly reduced compared with the group injected with dnMCAK (Figure 6J–K). These data strongly suggest that EZH2 can promote breast cancer metastasis through CCF. 

## 3. Discussion

CIN can lead to an increase in CCF [7]. Our results showed that CCF was down-regulated after HMGA1 was knocked down in MM-231 cells, while CCF was not up-regulated after HMGA1 was overexpressed in MCF-7 cells. It has been reported that HMGA1 can cause high CIN in tumor cells [38]. And HMGA1 participates in the formation of senescence-associated heterochromatin foci [34]. These results further indicate that HMGA1 is a necessary but not sufficient condition for CCF formation. Therefore, HMGA1 plays a key role in stabilizing CCF by the EZH2-HMGA1-USP7 complex. It has been reported that the micronuclear membrane can rupture due to a lack of lamin B1 [2], which is required for CCF formation. Our results show that the EZH2–HMGA1–USP7 complexes exist before and after micronuclear membrane rupture. We speculate that the heterochromatin structure of EZH2–HMGA1–USP7 complexes are likely to form CCF. However, it is unclear if CCF is allocated to progeny cells.

According to previous reports, TRIM14 recruits USP14 to promote cGAS activation [22]. Interestingly, the deubiquitinating enzyme USP27X also interacts with cGAS and cleaves the K48-linked polyubiquitinated chain from cGAS, thereby stabilizing the protein [21]. Our results show that activated cGAS can be colocalized with the deubiquitinase USP7 in breast cancer cells, and USP7 can stabilize activated cGAS in the cGAS–STING pathway by affecting the ubiquitination of cGAS. It has been demonstrated that USP7 and TRIM27 E3 ubiquitin ligase can exist in a complex. Therefore, we speculate that USP7 may regulate the ubiquitination of cGAS by interacting with TRIM27. We also show that the EZH2–HMGA1–USP7 complex can activate cGAS. As EZH2 is a methyltransferase, EZH2 and USP7 may regulate the levels of methylation and ubiquitination of cGAS, which leads to the production of inflammatory factors in tumor cells after the activation of cGAS. Further studies are needed to elucidate the mechanism of this action.

Since the EZH2-CCF-cGAS axis promotes breast cancer metastasis, EZH2i can inhibit breast cancer metastasis by regulating this pathway. USP7 can stabilize CCF, and the activation of cGAS is dependent on USP7. Therefore, we speculate that EZH2i combined with USP7i may inhibit breast cancer metastasis more effectively. Our research on the formation of CCF and the activation mechanism of cGAS has some new hints for the clinical treatment of breast cancer. Our research on the mechanism of CCF formation and cGAS activation has new implications for breast cancer treatment, suggesting that people may achieve the curative effect of breast cancer metastasis by targeting CCF with epi-modifying enzyme inhibitors.

Studies have found that the inflammatory factor in the tumor microenvironment can promote tumor metastasis in recent years. Since EZH2 promotes the formation of CCF and subsequently the production of inflammatory factors, inflammatory factors may promote metastasis by remodeling the pre-metastatic environment of the tumor into a pro-tumor environment. 

**Figure 7:**
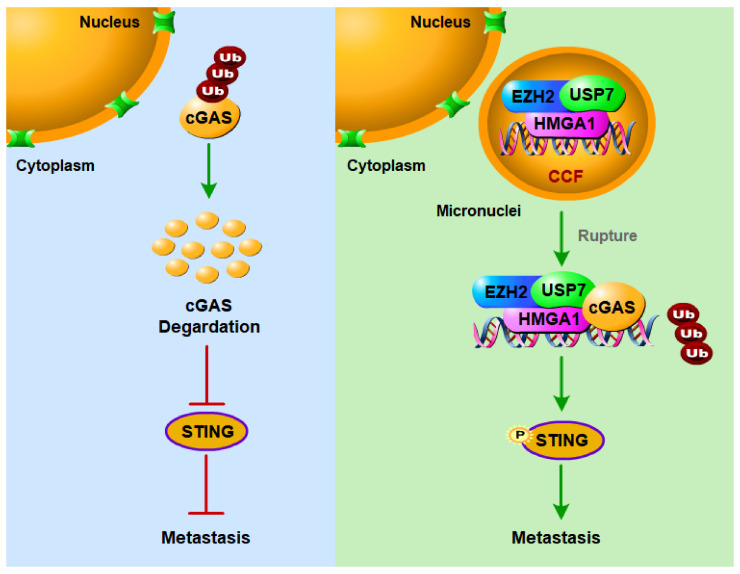
A proposed working model for CCF formation and activation of cGAS to promote breast cancer metastasis. In the highly metastatic breast cancer cells, EZH2 promotes the formation of CCF dependent on HMGA1, and the stability of EZH2 requires USP7. The activation of cGAS by EZH2 via CCF requires USP7 to deubiquitinate cGAS and stabilize cGAS. EZH2-CCF-cGAS axis can promote breast cancer metastasis, and targeting this axis with inhibitors can inhibit breast cancer metastasis.

In summary, we report that EZH2 promotes the formation of CCF in breast cancer cells and activates the cGAS–STING pathway to promote breast cancer metastasis. EZH2 can promote CCF formation in breast cancer cells, which depends on HMGA1, and the EZH2–HMGA1–USP7 complex stabilizes CCF. After the micronuclear membrane ruptures, USP7 in CCF can stabilize cGAS to activate the cGAS–STING pathway by reducing the ubiquitination of cGAS, thereby promoting breast cancer metastasis (Figure 7). Ultimately, this study provides evidence of a direct link between EZH2 and CCF–cGAS activation in tumor cells. It is also worth noting that the EZH2–CCF–cGAS axis promotes breast cancer progression, and the destruction of this axis by inhibitors weakens cancer cell migration and invasion, thus inhibiting breast cancer metastasis. This may lay the foundation for the development of new treatment strategies.

## 4. Materials and Methods

### 4.1. Cell Culture

Cell lines were obtained from the American Type Culture Collection (Manassas, VA, USA), where these cell lines were characterized by DNA fingerprinting and isozyme detection. Cells were immediately expanded and frozen so that they could be revived every 3–4 months. Cells were tested every 2 months to ensure that they were negative for mycoplasma contamination with the MycoA-lert Mycoplasma Detection Kit (LT07-218, Lonza, Basel, Switzerland). MCF-7 and MCF-7/ADR cells were maintained in RPMI-1640 medium supplemented with 10% Fetal Bovine Serum (VivaCell, Shanghai, China) and 10 μg/mL human recombinant insulin (Sigma-Aldrich, St. Louis, MO, USA). MM-231 cells were cultured in L-15 medium (Sigma-Aldrich) supplemented with 10% FBS. HEK-293T cells were cultured in Dulbecco’s modified Eagle medium (Sigma-Aldrich) supplemented with 10% FBS. Cell lines were cultured at 37 °C with 5% CO_2_, while MM-231 cells were cultured at 37 °C without CO_2_.

### 4.2. Plasmid and Short Hairpin RNA Transfections and Lentiviral Production

The following vectors were used in this study: pCDH-CMV-Flag-cGAS, pCDH-CMV-Flag-MCAK, pCDH-CMV-Flag-dnMCAK, pCDH-CMV-EZH2, pCDH-CMV-HA-cGAS, pWPXLD-EZH2, pCDH-CMV-3×Flag-USP7, and pCDH-CMV-3×Flag USP7 C223S. The control, EZH2#1, and EZH2#2 short hairpin RNA (siRNA) plasmids were constructed in the pLKO.1-puro backbone. The lentivirus packaging vectors used were psPAX2 and pMD2.G. The generation of lentivirus in 293T cells and the transfection of lentiviral constructs into recipient cell lines were performed following the manufacturer’s instructions (Invitrogen). siRNA was chemically synthesized by JiMa Company (Shanghai, China). The sequences are provided in Supplementary Table S1.

### 4.3. Reverse Transcription, PCR, and Real-Time PCR Analysis

Reverse transcription, PCR, and real-time PCR were performed as described [39]. Total RNA was extracted from cells using the Trizolreagent (Takara, Dalian, China) following the manufacturer’s instructions. The cDNA was generated using the Reverse Transcription System (Promega). The sequences of PCR primers were listed in Supplementary Table S2.

### 4.4. CCF Determination Experiment and Quantitative Analysis Method

The CCF marker H3K27me3 was used for immunofluorescence, and then the number of CCF in ≥200 cells were counted under an inverted microscope, and then the proportion of CCF was counted. 

### 4.5. Immunofluorescence

Immunofluorescence was performed as described [40]. Cell nuclei were counterstained with a 500 nM concentration of DAPI (Sigma). Photographs were taken using a confocal microscope (OLYMPUS). The Antibodies were listed in Supplementary Table S3. 

### 4.6. Western Blotting

Western blotting was performed as described [39]. Cells were lysed in 1×Laemmli sample buffer, and 5–20 µg of protein was resolved by SDS-PAGE followed by transfer onto PVDF membrane and probing with antibodies. The antibodies were listed in Supplementary Table S3.

### 4.7. Wound-Healing, Transwell Migration, and Invasion Assays

These experiments were performed as described before [41]. For the wound-healing assay, cells were plated in 6-well plates at a density of 1 × 10^6^ cell/well. A 10-μL pipette tip scratched cell layers, and the progression of migration was detected. In vitro cell migration and invasion assays were performed using Transwell chambers with 8.0-μm polyethylene terephthalate membrane (24-well inserts, Corning BioCoat, Cat.No.354166). For the migration assay, 2 × 10^4^ cells were seeded into the top chambers. For the invasion assay, 2 × 10^5^ cells were added to top chambers coated with Matrigel (BD Biosciences, Cat.No.356234). A basic medium was added to the top chambers, while a complete medium was added to the bottom wells to stimulate migration or invasion. After incubation, cells adhered to the lower surface of the membrane were stained with 0.1% crystal violet. 

### 4.8. Co-Immunoprecipitation

The co-IP assay was performed as described [39]. The antibodies were listed in Supplementary Table S3.

### 4.9. In Vivo Mouse Lung Metastasis Assay

A detailed description of the assay has been previously published [39]. In brief, stable MM-231-MCAK and MM-231-dnMCAK cells (1 × 10^6^ cells suspended in 150 L of PBS) were injected into the tail veins of female BALB/c nude mice (HFK Bioscience, Beijing, China) at 5 weeks of age. The intraperitoneal injection was started 1 week after the tumor was injected and administered every day for 2 weeks. Approximately 4 weeks later, the mice were sacrificed with euthanasia, and lung tissues were fixed in paraformaldehyde solution before counting the number of metastatic lesions. Thereafter, lung tissues were embedded in paraffin and processed for hematoxylin-eosin staining. All animal experiments were approved by the Animal Care Committee of the Northeast Normal University, Changchun, China.

### 4.10. Statistical Analysis

Results were compiled from at last three independent replicate experiments and presented as mean ± SD. The paired Student’s *t*-test (two-tailed) was used to calculate the significance of differences between groups. Data were considered significant at *p* < 0.05. Statistical analysis was conducted using GraphPad Prism software (GraphPad Software, La Jolla, CA, USA).

## Data Availability

The data presented in this study are available on request from the corresponding author.

## Supplementary Materials

Supplementary materials can be found at https://www.mdpi.com/article/10.3390/ijms23031788/s1.
