# Supplementary material for: EZH2–CCF–cGAS Axis Promotes Breast Cancer Metastasis
Source: Int J Mol Sci. 2022 Feb 4;23(3):1788. doi: 10.3390/ijms23031788 (PMC8836657; doi:10.3390/ijms23031788)
Supplement: Supplementary file 1 [file ijms-23-01788-s001.zip › Supplementary.pdf]

Supplementary

Figure S1 CCF ratio is higher in MM-231 breast cancer cells

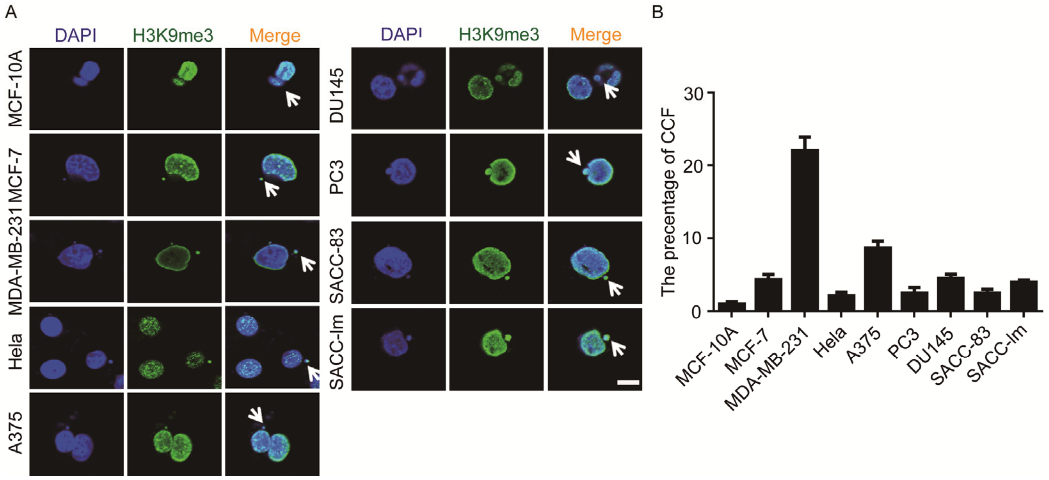

S1 CCF exists in different tumor cells. A-B MCF-10A, MCF-7, MM-231, HeLa, A375, DU145, PC3, SACC-83, SACC-lm cells were immunofluorescently stained with H3K9me3 to calculate the ratio of CCF, the arrow indicates CCF, Scalebars=10μm. Each experiment was repeated at least 3 times. Error bars, mean ± SD, **， $P < 0.01$； ***， $P < 0.001$.

Figure S2 EZH2 affects CCF formation in breast cancer cells.

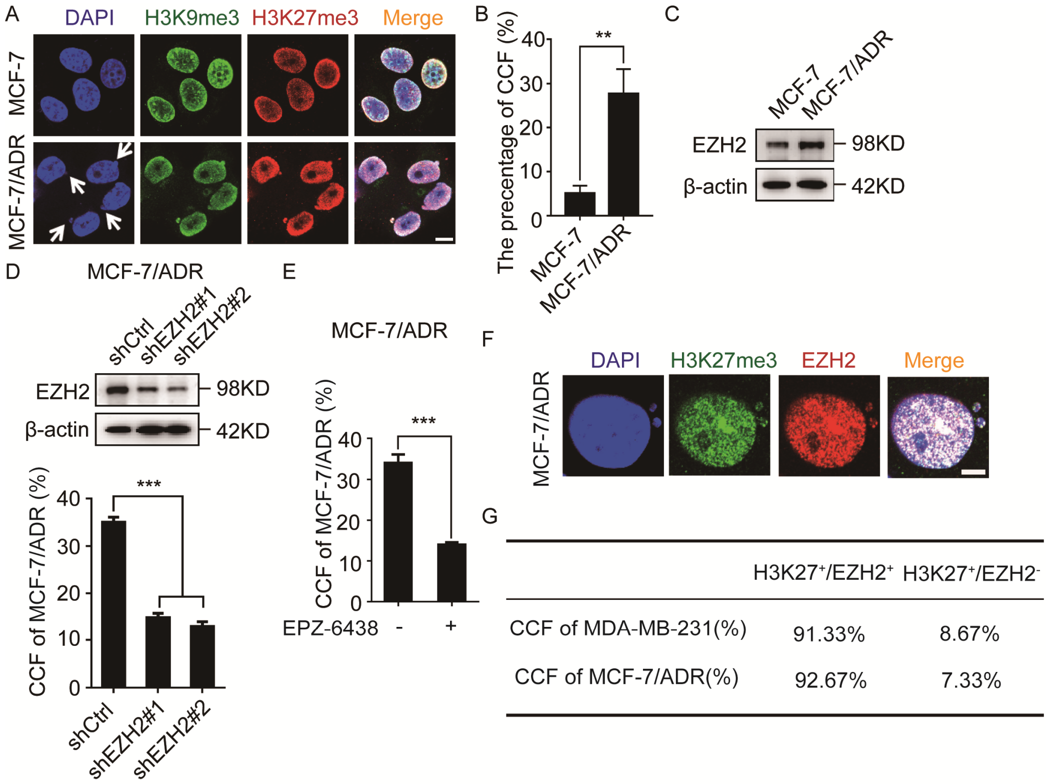

	H3K27⁺/EZH2⁺	H3K27⁺/EZH2⁻
CCF of MDA-MB-231(%)	91.33%	8.67%
CCF of MCF-7/ADR(%)	92.67%	7.33%

A-B MCF-7, MCF-7/ADR cells were immunofluorescently stained with H3K9me3 and H3K27me3 to calculate the ratio of CCF. CCF were indicated by arrows. Scale bars=10 μm. Western blotting was used to detect the EZH2 level in MCF-7, MCF-7/ADR **(C)**, MCF-7/ADR-shCtrl/MCF-7/ADR-shEZH2 cells **(D)**. The ratio of CCF was calculated by immunofluorescence in MCF-7/ADR-shCtrl/MCF-7/ADR-shEZH2 cells (D), MCF-7/ADR cells treated with EPZ-6438 (3 μM) for 72 h **(E)**. **F** EZH2, H3K27me3 were immunofluorescently stained in MCF-7/ADR cells to observe the co-localization of EZH2 with CCF. **G** The ratio of H3K27me3^{+}/EZH2^{+} and H3K27me3^{+}/EZH2^{-} cells in the CCF of MM-231 and MCF-7/ADR cells were calculated respectively. Scale bars=5 μm. Each experiment was repeated at least 3 times. Error bars, mean ± SD, **， $P < 0.01$；　***， $P < 0.001$.

Figure S3 EZH2 affects cGAS-STING pathway activation in breast cancer cells.

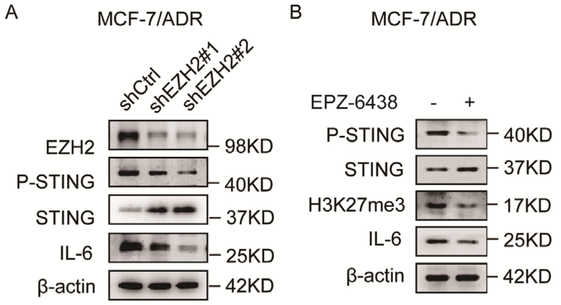

Western blotting was used to detect the P-STING, STING and IL-6 level in MCF-7/ADR-shCtrl/MCF-7/ADR-shEZH2 **(A)**, MM-231 cells treated with EPZ-6438 (3 μM) for 72 h **(B)**.

Figure S4 HMGA1 is a marker of CCF in breast cancer cells.

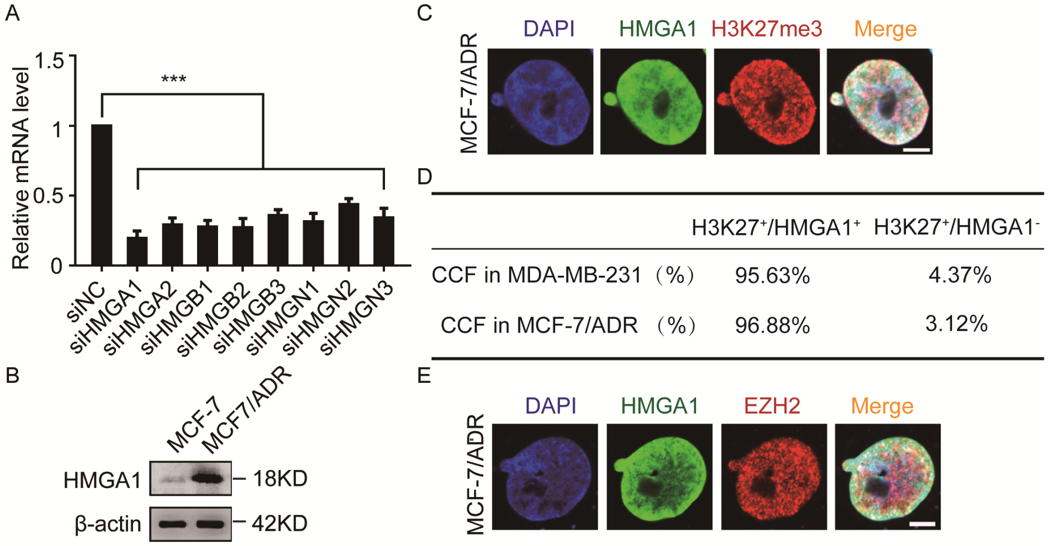

A siRNA is used to knock down HMG family proteins in MM-231 cells, and q-PCR is used to detect interference efficiency. **B** Western blotting was used to detect the HMGA1 level in MCF-7/ADR cells. **C** MCF-7/ADR cells were immunofluorescently stained with HMGA1 and H3K27me3 to observe the co-localization of HMGA1 and CCF, Scalebars=5 μm. **D** The ratio of H3K27me3^{+}/HMGA1^{+} and H3K27me3^{+}/HMGA1^{-} cells in the CCF of MM-231 and MCF-7/ADR cells were calculated respectively, Scalebars=5 μm. **E** MCF-7/ADR cells cells were immunofluorescently stained with HMGA1 and EZH2 to observe the co-localization of HMGA1 and EZH2, Scalebars=5 μm. Each experiment was repeated at least 3 times. Error bars, mean ± SD, **, $P < 0.01$; ***, $P < 0.001$.

Figure S5 EZH2 promotes breast cancer migration through CCF.

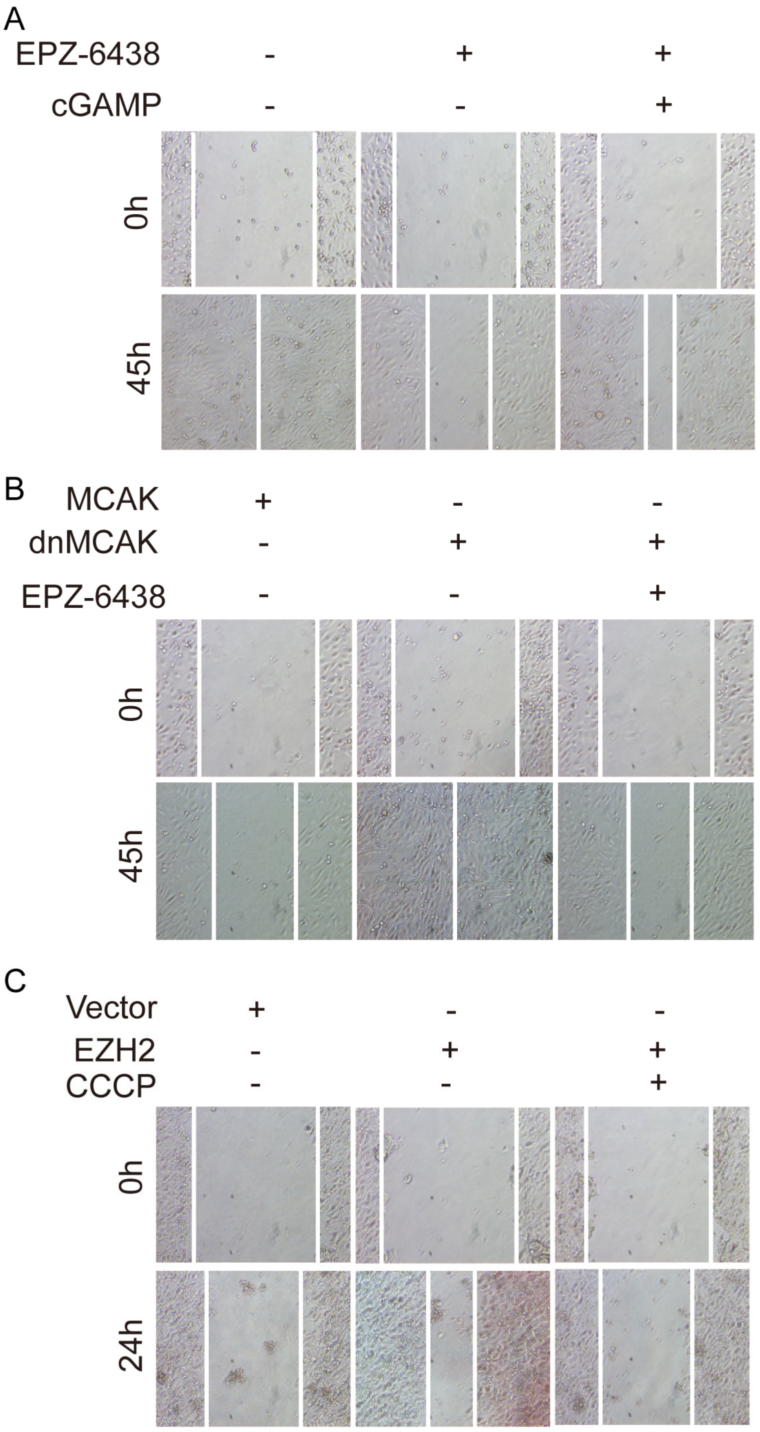

Wound-healing assay of MM-231 cells were treated with EPZ-6438 and cGAMP **(A)**, MM-231-dnMCAK cells treatment with EPZ-6438 **(B)**, MCF-7-EZH2 cells treatment with CCCP **(C)**.
